# Brain temporal dynamics correlate with clinical traits, spontaneous arm movements, and recovery in middle cerebral artery stroke

**DOI:** 10.3389/fnhum.2025.1617825

**Published:** 2025-10-20

**Authors:** Ziye Lei, Mengting Jiang, Yusi Wu, Hua Luo, Xiu Chen, Jianghai Ruan

**Affiliations:** ^1^Department of Neurology, Luzhou People's Hospital, Luzhou, China; ^2^Department of Neurology, The Affiliated Hospital of Southwest Medical University, Luzhou, China; ^3^Laboratory of Neurological Diseases and Brain Function, The Affiliated Hospital of Southwest Medical University, Luzhou, China

**Keywords:** acute ischemic stroke, electroencephalogram, microstate analysis, Actiwatch, middle cerebral artery stroke

## Abstract

**Objective:**

The purpose of this study was to look into the brain functional network changes and their possible correlations with clinical traits, spontaneous arm movements, and recovery in middle cerebral artery stroke.

**Methods:**

The study included 34 patients with acute cerebral infarction (CI) at middle artery and upper limb dyskinesia, as well as 50 healthy control (HC) participants. The spontaneous activity data of both upper limbs were recorded using a wrist activity recorder for 24 h. The Modified Rankin Scale (mRS) scales were then completed 90 days after the stroke onset. Resting-state EEG was acquired from both the CI and HC groups, and brain network features were examined using the microstate analysis. The correlations between microstates, spontaneous activity and clinical traits were investigated.

**Results:**

Compared with the HC group, the CI group had a higher MsB duration, lower MsC coverage and occurrence, and a decrease in both MsA and MsB to MsC as well as a decrease in MsC to MsA (*p* < 0.05); only the transition from MsC to MsB was enhanced; Reduced MsD coverage, occurrence, and duration when patients had larger FMA scores (*p* < 0.05); The MsB to MsA and MsD significantly correlated with FMA. Moreover, we found increased MsC coverage and duration, as well as the transition rates of MsA and MsD to MsC in those patients with mRS scores larger than one at 90 days after stroke (*p* < 0.05). Parts of Ms parameters involving MsA significantly correlated with mRS at 90 days after stroke.

**Conclusion:**

The dynamic balance of brain networks is altered when cerebral infarction occurs, and microstates offer a portion of the functional brain network foundation that allows us to recognize these alterations. These changes in temporal dynamic parameters effectively suggest the clinical traits and functional recovery in CI.

## 1 Introduction

Stroke is a group of cerebrovascular illnesses characterized by ischemic or hemorrhagic damage to brain tissue (GBD 2019 Stroke Collaborators, 2021). According to the China Stroke High-risk Population Screening and Intervention Program, an estimated 17.8 million adults in China experienced a stroke in 2020, with 3.4 million experiencing their first-ever stroke and another 2.3 million dying as a result (Tu and Wang, 2023). Ischemic stroke (IS) accounts for 5.2% of all deaths worldwide over five years (GBD 2015 Mortality and Causes of Death Collaborators, 2016; Fu et al., 2015). In clinical practice, the severity of stroke patients is typically assessed using clinical measures (Kasner, 2006), such as the National Institutes of Health stroke scale (NIHSS) (Yamal and Grotta, 2021), the modified rankin scale (mRS) (Haggag and Hodgson, 2022), the Barthel index (Liu et al., 2020), the Fugl-Meyer upper extremity exercise scale (FM-UE) (Kerimov et al., 2021), and others. In recent years, numerous criteria have been employed to investigate movement recovery following stroke (Schwarz et al., 2019). According to earlier research, spontaneous arm movements (Chen et al., 2022) on the affected side may decrease or stop entirely in the presence of dyskinesia before reappearing as a sign of recovery (Siekierka-Kleiser et al., 2006; Nakayama et al., 1994). Research in this area has received a lot of attention recent years, such as Mirror therapy in upper limb motor recovery (Nogueira et al., 2021), and research on therapies that have an impact on upper limb motor function and activities of daily living in the subacute and chronic phases of stroke, including virtual reality, robot-assisted therapy and telerehabilitation (Everard et al., 2022). In this study, we focus on publicly available wearable devices and associated features to monitor upper limb activity after stroke (Elgendi and Menon, 2019). Wearable gadgets, such as smart watches and wristbands, have given rise to wearable devices as prominent technologies (Ahmed et al., 2023).

A broadband electroencephalography (EEG) microstate technique is being used in an increasing number of clinical and cognitive neuroscience investigations to assess the electrical activity of large-scale cortical networks (Tarailis et al., 2024). EEG microstate analysis consists of grouping the spatial topographies of the sensor-space electric potentials (often referred to as “maps”) that are captured by EEG into a limited number of distinct clusters that, usually account for a significant portion of the data variation (Khanna et al., 2015; Michel and Koenig, 2018). Large-scale resting-state networks established by blood oxygen level-dependent signals are represented by microstates A, B, C, and D, which are typically categorized into four groups: visual networks, salience networks, auditory and vestibular system networks, and dorsal attentional networks (Britz et al., 2010; Seitzman et al., 2017). These groups represent various roles, including participation in speech processing within the auditory network (Damborská et al., 2019), reflection of vision (Vellante et al., 2020), involvement in sensory-motor information processing (Chu et al., 2020), overall cognition and emotion, and maintenance of attentional stability (D'Croz-Baron et al., 2021). In certain cases, the lesioned brain region can experience functional segregation and reorganization through modifications to the connections between other distant brain regions and changes in the function of surrounding normal brain regions (Hu et al., 2018), which helps to make up for the lost function in the damaged cortex (Hu et al., 2018). Because brain networks of spontaneous brain activity and microstates are closely related, microstate dynamics can partially represent motor abilities (e.g., function of the lower and upper limbs) (Spisak et al., 2020; Zhang et al., 2022).

In recent years, EEG microstates have gained increasing attention as a tool to probe brain network alterations after ischemic stroke in diagnosis (Hao et al., 2022; Lu et al., 2024; Lima et al., 2025), rehabilitation (Yu et al., 2023; Lv et al., 2025), and outcome prediction (Kong et al., 2025). Altered microstate dynamics have been linked to the cognitive function (Barzon et al., 2024), regions of lesions (Chen et al., 2025), and post-stroke level of consciousness (Wang F. et al., 2024). These findings suggest a potential role of EEG microstates as biomarkers of stroke in various aspects. While current evidence remains largely observational, more studies are still required to establish the clinical utility of microstates for diagnosis, prognosis, and therapy planning in stroke. The wearable Actiwatch is an important technology to record the spontaneous movements in stroke rehabilitation. It has been used to score the sleep impairments and motor recovery in post-stroke patients (Wang J. et al., 2024; Smith et al., 2024; Wang J. E. et al., 2024). Few studies combined the EEG microstate and actigraphy techniques to investigate the brain brain functional dynamics and spontaneous arm movements and their correlations with clinical traits after stroke.

In addition to providing a partial basis of the functional brain network for our identification of changes in spontaneous bilateral upper limb activity and its relationship with the brain network in the early recovery of CI patients, the goal of this study aimed to predict the early recovery and determine the severity of CI patients by combining EEG microstate analysis and spontaneous bilateral upper limb activity with clinical scale scores.

## 2 Materials and methods

### 2.1 Participants

From January 2022 to October 2023, 34 patients with first-ever acute MCA ischemic stroke and upper limb dyskinesia in addition to 50 healthy elderly volunteers were recruited from the Department of Neurology, the Affiliated Hospital of Southwest Medical University. This study included a total of 84 participants. This study was approved by the Ethics Committee of the Affiliated Hospital of Southwest Medical University, and informed consent was obtained from all participants and their families.

The inclusion criteria for the stroke patients were as follows: (1) patients with acute cerebral infarction hemiplegia diagnosed with acute stage ischemic stroke within 7 days of ischemia by a neurologist according to the *Chinese guidelines for diagnosis and treatment of acute ischemic stroke 2018* (Tu and Wang, 2023; Chinese Society of Neurology, Chinese Stroke Society, 2018); (2) imaging examination after admission suggesting infarction in the blood-supplying area of the MCA; (3) Aged 30–80 years old; (4) prior to this stroke, without other diseases affecting upper limb function; (5) right-handedness. (6) no other neurological or mental illnesses.

The inclusion criteria for the healthy elderly group were: (1) with no history of diabetes, hypertension, cardiovascular and cerebrovascular diseases, or mental illness; (2) cranial magnetic resonance scan showing no definite brain lesions; (3) right-handedness; (4) Aged 30–80 years old.

Exclusion criteria included history of other severe neuropsychiatric diseases and considerable EEG data interference: (1) cardiogenic or other causes of non-primary cerebral infarction; (2) severe heart, kidney, severe malnutrition, or immune system disease; (3) hemorrhagic stroke; (4) tumor; (5) patients with thrombolytic therapy in the acute phase; (6) pregnant patients; and (7) with other neurological or mental illnesses.

The criteria for participant deletion or discontinuation were as follows: (1) Patients who had obvious head movement during EEG acquisition and did not cooperate with clinical scale assessments; (2) Patients who requested automatic withdrawal; (3) Patients who were lost to outpatient follow-up; (4) Patients who experienced new stroke during early rehabilitation.

The patients were followed up after a 90-day recovery period, and the review process was performed by a specialized neurological physician to refine the mRS scores.

### 2.2 Clinical trait assessments

The participants’ demographic data were collected. Fugl-Meyer Assessment (FMA) for upper extremity, NIHSS, mRS, Montreal Cognitive Assessment (MoCA) (Carson et al., 2018), and Mini-Mental State Examination (MMSE) (Wang G. et al., 2022) scores were assessed by experienced neurologists within 7 days of stroke (acute phase) in patients with MCA infarction using a uniform assessment process. After about 90 days later, patients were followed up in an outpatient clinic, where the modified rankin scale (mRS) scores were refined by a specialized neurologist.

### 2.3 Actiwatch

The ware, a small light-weight gadget the size of wrist-watch, synchronously recorded movements in all three dimensions with movement-sensitive sensors (0.01 gravity to 8 gravity). The patients were introduced to wear the Actiwatch (MotionWatch, MW 8, CamNtech Inc. UK) on both wrists of the upper limbs for 24 h. The data were loaded and binned into minutes with Motionware software (v1.2.1, CamNtech Inc. UK). Then, we calculated the spontaneous activity parameters of upper limb activity in patients, including the bilateral upper extremity activity coordination index (*r*), the upper limb activity ratio (ULAR, %), and duration of moderate activity level (minutes). The moderate activity level was defined as the counts per minute above than 500. The ULAR were defined as the movement counts of affected arm divided by the contralateral healthy side (Equation 1).


(1) ULAR=Maffec.Mcontra.∗100%

The ULAR indicated the percentage of upper limb activity ratio, M_affec._ indicated the movements of the affected arm, and M_contra._ indicated the movements of the contralateral unaffected arm.

### 2.4 EEG recording

All patients completed EEG recordings for approximately 20 min. All participants were asked to remove scalp dirt and degrease with 75% alcohol before the examination; sit in a semi-isolated, temperature-appropriate room; stay awake; close their eyes; relax; and minimize their muscle activity during EEG collection. The acquisition instrument was an Italian EB Neuro EEG instrument, with a sampling rate of 500 Hz., and an impedance controlled below 10 kΩ. Scalp electrodes were installed according to the international 10–20 system developed by the International Federation of Clinical Neurophysiology. The scalp electrodes were placed in bilateral prefrontal pole (Fp1, Fp2), frontal pole (F3, F4), anterior temporal (F7, F8), middle temporal (T3, T4), posterior temporal (T5, T6), central region (C3, C4), parietal pole (P3, P4), occipital pole (O1, O2), parietal scalp (Cz), and ear electrodes A1 and A2 were used as reference electrodes.

### 2.5 EEG preprocessing

The EEG data were pre-processed with the Matlab based toolbox EEGlab (v13.6.5, http://sccn.ucsd.edu). The steps of pre-processing was similar with our previous studies (Zhou et al., 2023; Tan et al., 2024). Briefly, the original data were exported into the European Data Format and imported into EEGlab. Then the locations of the electrodes were assigned. The EEG data were notch-filtered at 50 Hz to decline the possible current noise. Subsequently, the EEGLAB plug-in Automatic Artifacts Removal (AAR)^1^ was employed to automatically correct ocular and myogenic artifacts. The AAR algorithm leverages blind source separation (BSS) combined with fast ICA algorithm method to isolate and remove electrooculogram components, which were identified based on their low fractal dimension. A similar algorithm was applied to suppress muscle artifacts. Channels exhibiting excessive noise were labelled as “bad” if their standard deviation (SD) exceeded a threshold of 4. Datasets with more than two bad channels were excluded from further analysis. Identified bad channels were then reconstructed via spherical interpolation. After that, the data were bandpass-filtered (1–45 Hz) using a zero-phase Butterworth filter implemented in MATLAB (functions filtfilt and butter). All EEG signals were re-referenced to the average reference. Finally, we selected five 10-s-epochs of EEG in the resting state with eyes closed for each participant for subsequent analysis. All preprocessing steps were executed using a custom MATLAB-based pipeline (R2016a, The MathWorks Inc.).

### 2.6 Microstate analysis

The preprocessed data were imported through the LORETA-Key tool (v20190617, Bain-heart: KEY Institute in Zurich, Switzerland, www.uzh.ch/keyinst/loreta) and then subjected to microstate analysis. First the Global Field Potential (GFP) of each time sample point were calculated (Lehmann et al., 1987) with the following Equation 2:


(2) $$\mathrm{GFP}(t)=\sqrt{\frac{\Sigma_{i=1}^{n}{\left[\mathrm{vi}(t)-\bar{v}(t)\right]}^{2}}{n}}$$

where n represents the total number of electrodes, vi(t) is the potential of the i-th electrode at time t. vi(t) is the mean of the instantaneous potentials across the electrodes. Because of the stability of the topography, the topography at the instantaneous maximum point of GFP were employed to present the surrounding topography for analysis (Cao et al., 2023). Then, the main primary topographic maps were classified by k-means clustering method (Pascual-Marqui et al., 1995). The polarity of the topographical map was ignored. According to our previous studies (Zhang et al., 2023; Zhou et al., 2023), We selected four canonical microstates due to their well-established neurophysiological relevance to human mental state and reliability. The individual maps for each participant were extracted and the mean Ms maps were calculated for each group. After that, the recognized individual maps were back-fitted across groups using their group mean maps. The labels corresponding to the original topographic map and Ms. were labeled as A, B, C, D in accordance with their tomographic distributions. Finally, the subsequent parameters were derived for each microstate at various time intervals (Khanna et al., 2015): (1) Coverage: percentage of the analyzed time occupied by a specific microstate (Ms); (2) Duration: the average duration of each microstate; (3) Occurrence: i.e., the average number of times per second that the Ms. occurs; and (4) Transition: the probability that one type of Ms. transfers to another.

### 2.7 Subgroup and correlation analyses

To further explore the changes of spontaneous activity and microstates after CI, we defined several subgroups, such as FMA ≤ 55 and FMA > 55, moderate activity time above >150 min and ≤150 min, bilateral upper limb coordination coefficients *r* > 0.62 and ≤0.62, mRS after 90 days ≤1 and >1, and ULAR >30% and ≤30%. In addition, correlations between clinical trait metrics including spontaneous upper-limb activity, clinically relevant scales such as NIHSS, FMA, mRS, MoCA, MMSE and Ms parameters were examined.

### 2.8 Statistical methods

A generalized linear model (GLM) with age and sex as covariates were used to decline the possible influences on results. Group and subgroup differences in the microstate parameters and parameters of arm spontaneous activity movements were explored using a two-sample *t*-test. The statistical significance level was set to *p* < 0.05. False discovery rate (FDR) correction was applied to correct for multiple comparisons. All the tests were carried out using MATLAB (R2016a, The MathWorks Inc.). The effect size between the two groups was described by calculating Cohen’s *d* value.

## 3 Results

Thirty-four stroke patients who met the inclusion and exclusion criteria were included in the acute CI group, while 50 individuals were included in the HC group. There were no significant differences in sex and age between the two groups (Table 1).

**Table 1:** Demographics of CI group and HC group included in this study.

Items	CI *n* = 34	HC *n* = 50	$x^{2}∕t$	*p*
Sex (male/female)	21/13	25/25	0.706	0.401^a^
Age (mean ± SD)	62.44 ± 9.76	59.58 ± 9.15	1.369	0.175^b^
Right hand paralysis cases [case (%)]	20 (58.8%)	\	\	\
NIHSS (mean ± SD)	6.53 ± 3.40	\	\	\
Onset time (hours, mean ± SD)	94.24 ± 69.75	\	\	\
FMA of affected arm (mean ± SD)	50.12 ± 20.30	\	\	\
MoCA (mean ± SD)	19.60 ± 5.54	\	\	\
MMSE (mean ± SD)	21.44 ± 4.38	\	\	\
LUA-MS (mean ± SD)	3.59 ± 1.69	\	\	\
RUA-MS (mean ± SD)	4.18 ± 1.29	\	\	\
mRS at 90 days (mean ± SD)	1.68 ± 1.00	\	\	\

CI, acute cerebral infarction group. HC, healthy control group; SD, standard deviation. NIHSS, National Institutes of Health stroke scale; mRS, modified Rankin scale; FMA, Fugl-Meyer Assessment; MoCA, Montreal Cognitive Assessment; MMSE, Mini-Mental State Examination; LUA-MS, left upper arm muscle strength; RUA-MS, right upper arm muscle strength.

a Chi-square test.

b two sample *t*-test.

**Figure 1:**
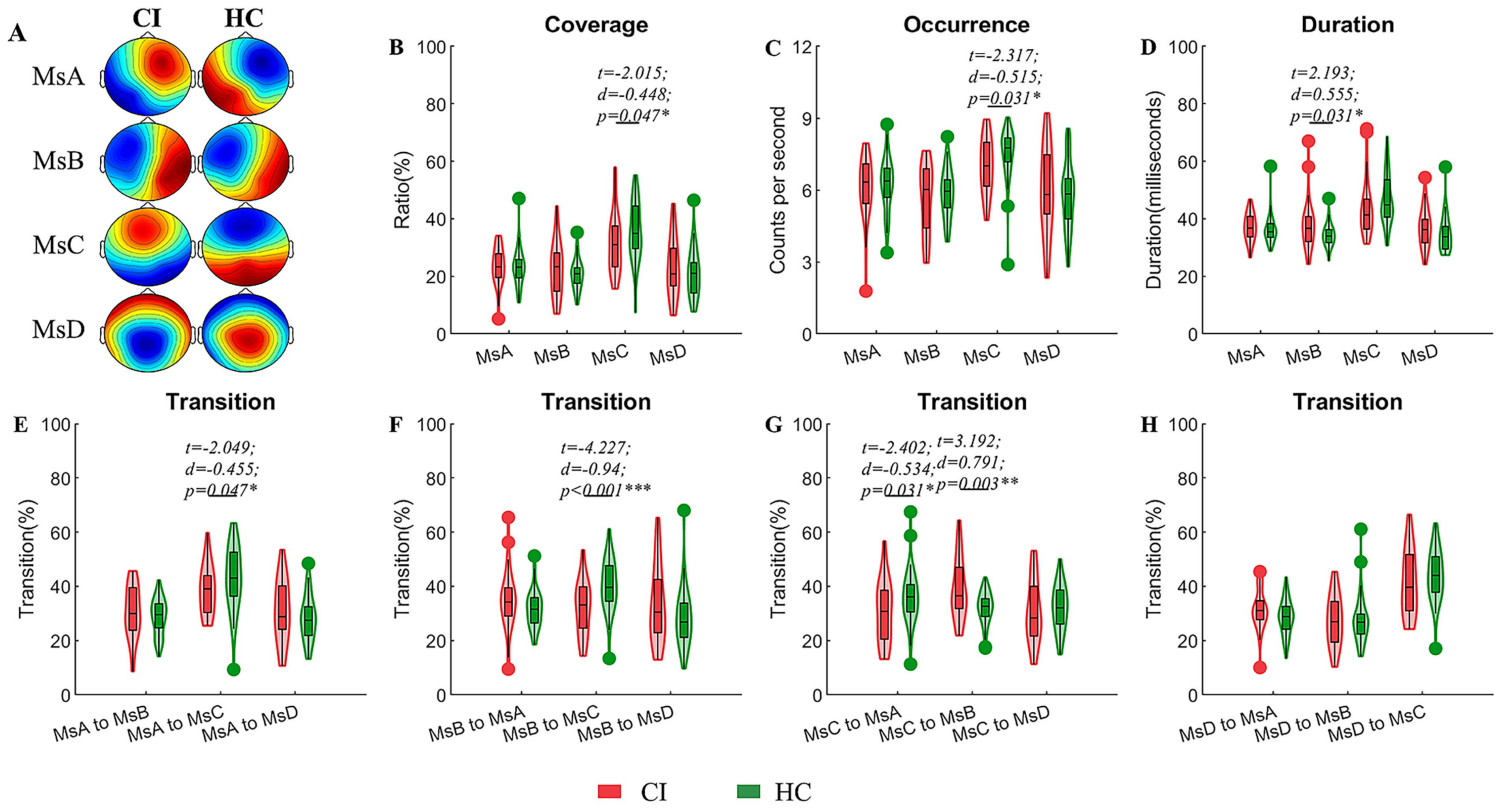
Comparisons of temporal dynamic parameters between the CI and HC groups. **(A)** EEG topographic maps for the four microstates of CI and HC groups; **(B–D)** coverage, occurrence, duration of microstates; **(E–H)** transition ratios between individual microstates. A generalized linear model (GLM) with age and sex as covariates were used to decline their possible impacts on the results. Two sample *t*-test with FDR corrections were used in these comparisons. CI, cerebral infraction; HC, healthy control; *d*, the effect size Cohen’s *d*. *p < 0.05; **p < 0.01; ***p < 0.001.

The CI group had a higher duration of MsB (*t* = 2.193, *d* = 0.555, *p* = 0.031) and lower coverage and occurrence of MsC (*t* = −2.015, *d* = −0.448, *p* = 0.047; *t* = −2.317, *d* = −0.515, *p* = 0.031) in comparison to the healthy group. In terms of microstate transition, the CI group saw a decrease in both MsA and MsB to MsC as well as a decrease in MsC to MsA; only the transition from MsC to MsB was enhanced (Figure 1).

**Figure 2:**
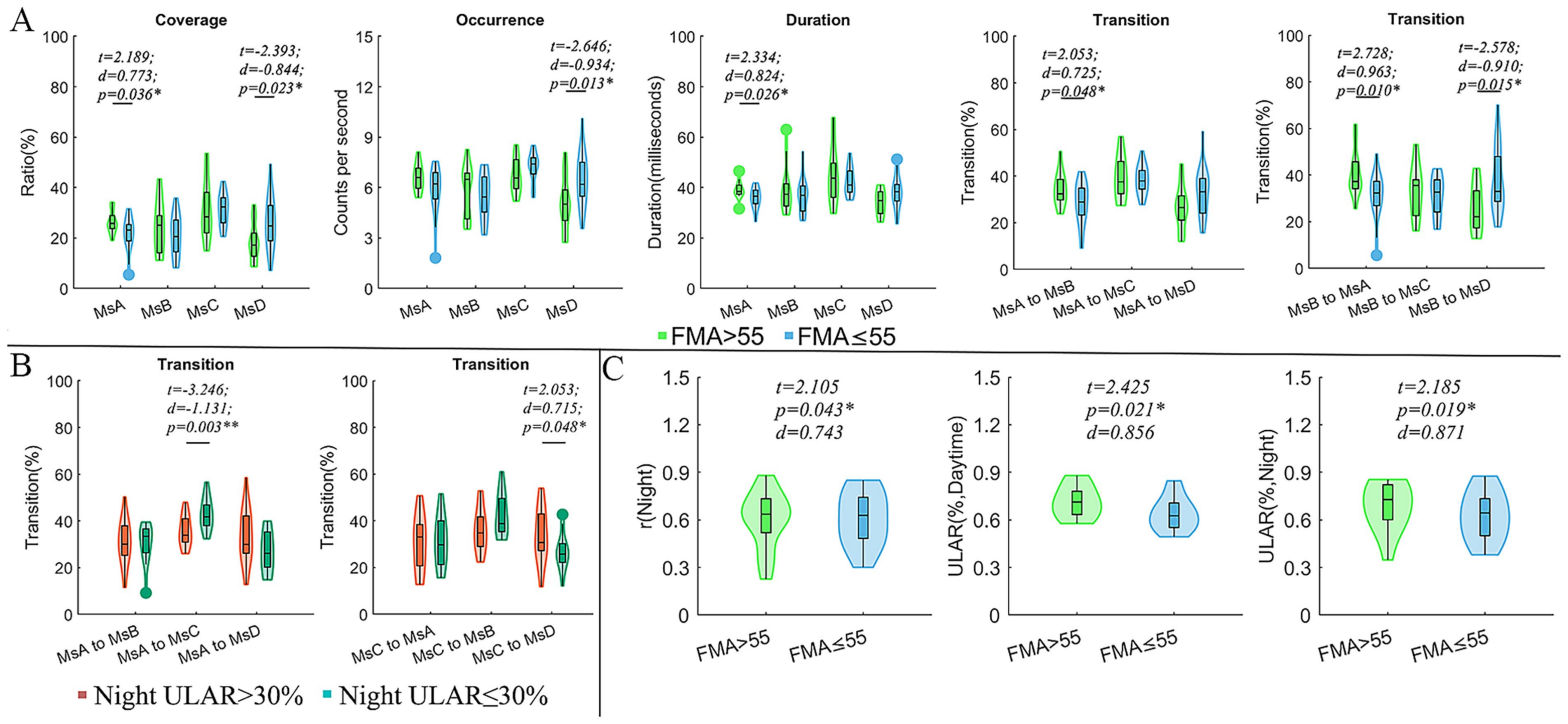
Comparisons of microstate parameters and actigraphy parameters between subgroups. **(A)** Comparison of microstate parameters between subgroups divided by FMA; **(B)** Comparison of microstate parameters between subgroups divided by ULAR in the night; **(C)** Comparison of actigraphy parameters between subgroups divided by FMA; In these comparisons, the age, sex, lesion hemisphere, NIHSS, Onset time and MoCA were included as covariates to exclude their possible effects on the results. FMA, the Fugl-Meyer upper extremity exercise scale; mRS, the Modified Rankin Scale; ULAR, the upper limb activity ratio; *t*, *t* values of two sample *t*-test; *d*, the effect size Cohen’s *d*. *p < 0.05; **p < 0.01.

**Figure 3:**
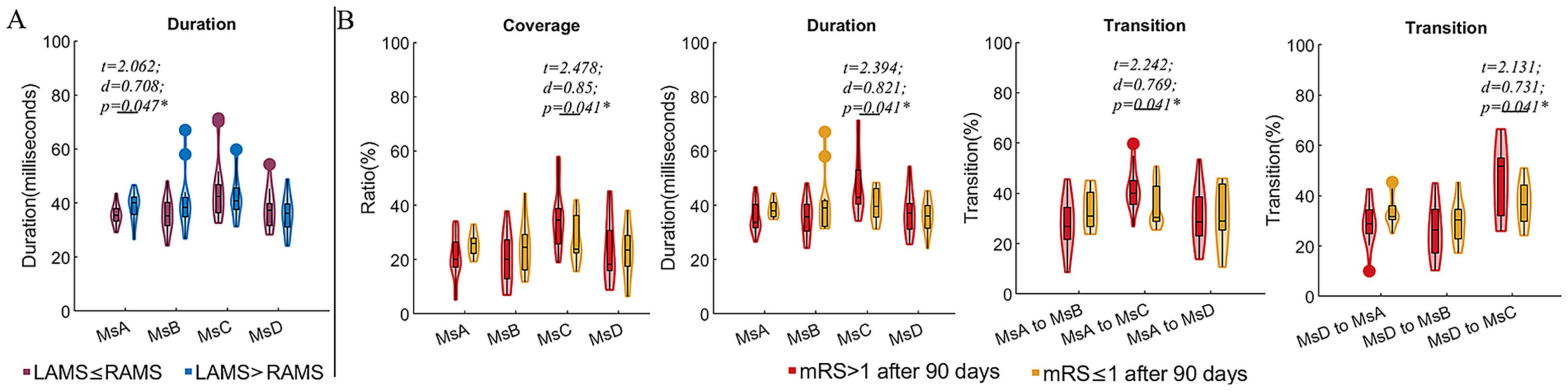
Comparison of microstate parameters between subgroups divided by arm muscle strength and mRS after 90 days of stroke onset. **(A)** Comparison of microstate parameters between subgroups with LAMS ≤RAMS and LAMS>RAMS. **(B)** Comparison of microstate parameters between subgroups divided by mRS after 90 days of stroke onset. Note: LAMS, left arm muscle strength; RAMS, right arm muscle strength; Ms, microstates; *t*, *t* values of two sample t-test; *d*, the effect size Cohen’s *d*. *p < 0.05.

Compared to CI patients with FMA scores ≤55, those with FMA > 55 exhibited reduced coverage and occurrence of MsD (*t* = −2.393, *d* = −0.844, *p* = 0.023; *t* = −2.646, *d* = −0.0.934, *p* = 0.013), as well as decreased coverage and duration of MsA (*t* = 2.189, *d* = 0.773, *p* = 0.036; *t* = 2.334, *d* = 0.824, *p* = 0.026). Additionally, the FMA > 55 subgroup exhibited higher transition probabilities between MsA and MsB (*t* = 2.053, *d* = 0.725, *p* = 0.048; *t* = 2.728, *d* = 0.963, *p* = 0.010), but a significantly lower transition probability from MsB to MsD (*t* = −2.578, *d* = −0.910, *p* = 0.015). For the spontaneous arm movement parameters, the CI patients with FMA > 55 exhibited increased night r (*t* = 2.105, *d* = 0.743, *p* = 0.043) and ULAR in both daytime (*t* = 2.425, *d* = 0.856, *p* = 0.021) and night (*t* = 2.185, *d* = 0.871, *p* = 0.019) (Figure 2). Moreover, we divided the subgroups by the night ULAR. Compared to those CI patients with ULAR≤30%, those with night ULAR>30% showed a reduced transition ratio from MsA to MsC (*t* = −3.246, *d* = −1.131, *p* = 0.003) while an increased transition ratio from MsC to MsB (*t* = 2.053, *d* = 0.715, *p* = 0.048) (Figure 2). Moreover, the duration of MsA decreased in the CI patients with left arm muscle strength (LAMS) ≤ right arm muscle strength (RAMS) than those patients with LAMS>RAMS (*t* = 2.062, *d* = 0.708, *p* = 0.047). In the subgroup with mRS scores >1 after 90 days of stroke onset, there was an increase in MsC coverage (*t* = 2.478, *d* = 0.850, *p* = 0.041) and duration (*t* = 2.394, *d* = 0.821, *p* = 0.041), as well as an increase in the transition rates from MsA and MsD to MsC transition (*t* = 2.242, *d* = 0.796, *p* = 0.041; *t* = 2.131, *d* = 0.731, *p* = 0.041) (Figure 3).

**Figure 4:**
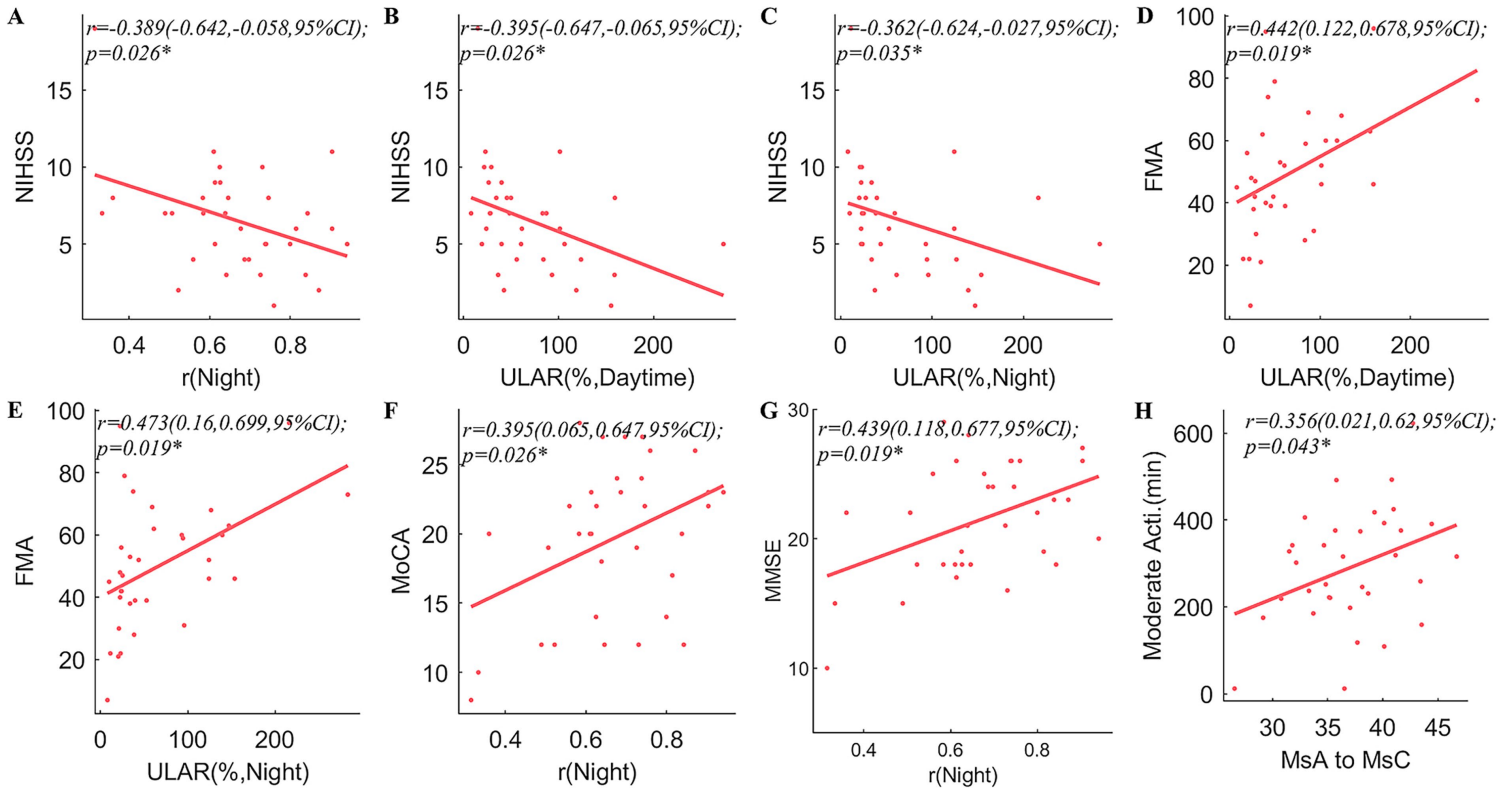
Correlation analyses between arm spontaneous activity and clinical scales and microstates. correlations between NIHSS and arm spontaneous activity parameters including *r* in the night **(A)**, ULAR at daytime **(B)**, and ULAR in the night **(C)**; **(D,E)** correlation between FMA and ULAR at Daytime **(D)**, and ULAR in the night **(E)**; correlations between bimanual activity correlation coefficients in the night and MoCA **(F)**, and MMSE **(G)**; Correlations between the transition of MsA to MsC and time spent with limbs above a moderate level of activity **(H)**. NIHSS, National Institutes of Health Stroke Scale; FMA, Fugl-Meyer Assessment; ULAR, Upper Limb Activity Ratio of the Affected Side to the Healthy Side; MoCA, Montreal Cognitive Assessment; MMSE, Mini-Mental State Examination; Ms, microstate; *r*, Pearson’s correlation coefficient. CI, confidence interval; *p < 0.05.

**Figure 5:**
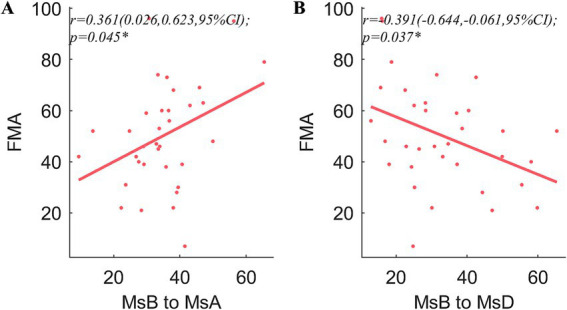
Correlation analysis of the microstates and clinical traits. FMA is significantly correlated with the transition ratios from MsB to MsA **(A)** and MsB to MsD **(B)**. The other scales showed no significant correlations were not shown in the figure. *r*, Pearson’s correlation coefficient. CI, confidence interval; *p < 0.05.

Pearson’s coefficient of bilateral upper extremity coordination, night *r*, was positively correlated with MMSE (*r* = 0.439, *p* = 0.019) and MoCA (*r* = 0.395, *p* = 0.026). In both the daytime and the night, the ULAR had a positive correlation with FMA (*r* = 0.442, *p* = 0.019; *r* = 0.473, *p* = 0.019) and an inverse correlation with NIHSS (*r* = −0.395, *p* = 0.026; *r* = −0.362, *p* = 0.035). And the night *r* was negatively correlated with NIHSS (*r* = −0.389, *p* = 0.026). In addition, the rates of MsA to MsC conversion were positively linked with time spent above moderate levels of activity (*r* = 0.356, *p* = 0.043) (Figure 4). The conversion rate of MsB to MsA was favorably correlated with FMA (*r* = 0.361, *p* = 0.045), while the transition of MsB to MsD was adversely correlated (*r* = −0.391, *p* = 0.037) (Figure 5).

Spearman’s correlation analyses found that mRS after 90 days were negatively correlated with the coverage (*r*_s_ = −0.365, *p* = 0.034) and duration (*r*_s_ = −0.397, *p* = 0.020) of MsA, and the transition probabilities from MsD to MsA (*r*_s_ = −0.420, *p* = 0.013). However, mRS after 90 days were positively related to the coverage (*r*_s_ = 0.462, *p* = 0.006) and duration of MsC (*r*_s_ = 0.498, *p* = 0.003), the transition probabilities from MsA to MsC (*r*_s_ = 0.454, *p* = 0.007) and MsD to MsC (*r*_s_ = 0.355, *p* = 0.039).

## 4 Discussion

Using microstate analysis, we were able to extract the brain network from the EEG recordings of the healthy group and the case group. Meanwhile, we used Actiwatch to collect 24-h continuous bilateral upper limb activity data of CI patients, combined with clinical scales commonly used to assess the severity of the stroke, to evaluate the characteristics and efficacy of spontaneous activity in early recovery of CI patients. The current study was committed to comprehending the alterations in the functional brain network in patients with middle cerebral artery strokes and their potential correlation with clinical features, spontaneous arm movements, and recovery, even though many research hotspots in the last 2 years have focused on changes in brain dynamics following brain injury (Comsa et al., 2019; Bonkhoff et al., 2020; Bai et al., 2021).

Numerous recent investigations (Gan et al., 2023; Li et al., 2022; Schiller et al., 2021) have established that the brain network change is a sophisticated process, which is transformed and regulated in each of the four microstates in various situations. We noted the coverage and occurrence of MsC in patients with cerebral infarction are decreased, while the duration of MsB is elevated, which is behind the final presentation of the results due to the decrease in the transition of MsA and MsB to MsC, MsC to MsA, and the increase in the transition of MsC to MsB. These four network systems: auditory network, visual network, salience network, dorsal attention network, correspond to microstate A, B, C, and D, which are large-scale resting-state networks established by blood oxygen level-dependent signals (Seitzman et al., 2017; Schulz et al., 2021). These clusters in turn represent roles that: participate in the auditory network related to speech processing and reflect vision (Schulz et al., 2021), participate in sensory-motor information processing and overall cognition and emotion (Seeley et al., 2007), and maintain attentional stability (Katayama et al., 2007). Ultimately the result of the increase of the salient network and the decrease of the visual network in the CI group occurs due to the loss of compensation, which would indicate that the patients with acute cerebral infarction have a decrease in the visual processing function but a compensatory increase in the involvement of sensory-motor information processing and the overall cognitive and affective abilities. This differs somewhat from the findings of Zappasodi et al. (2017). Normally we think that patients with CI may be at risk for vascular cognitive dysfunction (Rost et al., 2022; Rundek et al., 2022).

Interestingly, the mRS score after 90 days was positively correlated with the duration and coverage of MsC, as well as with the transition from MsA to MsC and MsD to MsC. Yet, it was negatively correlated with the transition of MsA to MsB and the occurrence of MsB. MsC is positively linked with activation in the bilateral temporal gyrus, posterior cingulate cortex, and insula segments, and it represents alterations in the activity of salient network. Brain damage, such as a stroke, results in extensive structural and functional network failure in addition to behavioral abnormalities (Sebastian-Romagosa et al., 2020). The mRS is a global scale of disability or dependence in daily activities (Rankin, 1957). Prior research has demonstrated a correlation between motor scores and EEG characteristics of spontaneous brain activity such as functional connectivity (Salvalaggio et al., 2020; Hoshino et al., 2021). The present study may suggest the presence of a compensatory elevation of the salience network in patients with a higher mRS, i.e., the more severe the paralysis and the worse the prognosis, which is achieved by an increase in the transition of the auditory network and the dorsal attentional network towards it. MsC and MsD are in a condition of dynamic equilibrium (Santarnecchi et al., 2017). The degree to which equilibrium is upset following a stroke varies based on how severe the stroke motor dysfunction (Wang Z. et al., 2022).

The duration, coverage, and occurrence of MsD were decreased in the subgroup with FMA > 55 compared to patients with FMA ≤ 55 and this loss of compensation was the result of a significant decrease in transition from the MsB to MsD and an increase in the MsB to MsA, but ultimately no significant change in MsB and MsA. Patients with more severe hemiparesis showed improved ability to maintain attentional stability. Although attention was improved in patients with more severe hemiplegia, the auditory network related to speech processing was still significantly reduced. The mechanism behind this may be explained by the compensatory theory of increased and decreased functional connectivity (FC) between brain networks in post-stroke patients (Baldassarre et al., 2016).

The patients with higher mRS scores at 90 days, with a higher ability to engage in sensorimotor information processing and overall cognition and emotion, which is consistent with the findings of our line correlation analyses described above. Patients with a night ULAR (activity ratio of the affected healthy side at night) ≤ 30% were more severely paralyzed, with an increased rate of MsA-to-MsC transition and an elevated rate of MsC-to-MsB transition, but ultimately there was no functional imbalance of the four attentional networks. Based on functional compensation, if some of the patient’s functions can be preserved and maintained at a steady state, perhaps as a result of compensating for the reduced performance of the original function through other functions (Hall et al., 2021).

ULAR was higher at both daytime and night in patients with FMA > 55 scores, The FMA scale developed as an evaluative measure of recovery from hemiplegic stroke, It is divided into 5 domains: motor function, sensory function, balance, joint range of motion, and joint pain (Gladstone et al., 2002). The Actiwatch is a motion sensor that uses electronics to detect changes in velocity or acceleration. It is wearable on the wrist to track movement of the body (Lee and Tse, 2019). To some extent, both FMA and ULAR extracted via Actiwatch can reflect the process of dynamic motor recovery in stroke patients, unlike the evaluation of motor recovery by Reynolds et al. (1958), Brunnstrom (1966) which exists only qualitatively. Another interesting finding is that the duration of the MsA is trending downward in patients with left-handed paralysis, i.e., patients with left-handed paralysis are involved in a spontaneous decline in the auditory network that can be associated with speech processing. Previous research comparing stroke patients to controls revealed varying times for microstates C and D following left- and right-sided lesions (C lower than D in left-sided lesions, and D lower than C in right-sided lesions) (Zappasodi et al., 2017).

Last, in the correlation analysis, we found that ULAR during the day and at night was inversely proportional to the NIHSS score and positively proportional to the FMA, it represents that ULAR has the same cue significance as the clinical scales. The higher the ULAR was, the lower the NIHSS score was, and the higher the FMA score was, the less severe the paralysis was in the patients. The NIHSS uses basic objective binary outcomes, which are insufficient to reflect the dynamic process of motor recovery and are less likely to detect changes in response to specific treatments (Gladstone et al., 2002). More objective methods of assessing stroke have been a hot topic in recent years, and Bezuidenhout et al. (2022) have shown that accelerometers, for example, can assess stroke severity in patients, which is consistent with our study. The r-night index of bilateral upper extremity coordination showed a similar pattern, with r-night being inversely proportional to the NIHSS score and positively proportional to the MoCA and MMSE scores. Shih et al. (2023) have demonstrated the impact of stroke on limb coordination. This may suggest that the more coordinated the bilateral upper extremity activities, the less paralyzed and less cognitively impaired. Szameitat et al. (2012) revealed the first demonstration of the neuroanatomical correlates of imagined bimanual coordination skills. Building on the earlier work of Puttemans et al. (2005), they predicted and provided evidence that connection would alter rather than selectively increasing neuronal activity in a particular cortical region of interest.

The study of brain network characteristics and bilateral upper extremity spontaneous activity in early recovery of CI patients is very novel, suggesting the mechanism of brain network compensation in acute ischemic stroke and how the degree of paralysis affects the strength of brain network compensation, and verifying that the bilateral upper extremity spontaneous activity can be used as an indicator for observing early prognosis of the patients, which provides a new diagnostic and therapeutic idea for the patients with CI.

There are some limitations in this study: The small sample size, along with the exclusion of patients treated with thrombolysis or thrombectomy, limits the generalizability of the findings to the broader stroke population. The current findings are exploratory and warrant validation in larger and more diverse cohorts. In addition, although microstate features have been proposed as candidate biomarkers, our analyses were not designed to test predictive performance, and clinical utility remains to be determined. In future, it is necessary to expand the sample size and the scope of patient data collection in longitudinal studies to provide a better and more objective assessment index for the prediction of acute cerebral infarction at the early clinical stage.

## 5 Conclusion

In this study, we collected resting-state EEG from patients with acute middle cerebral artery infarction and healthy elderly group and extracted brain network parameters by microstate analysis, which showed that the occurrence of CI is accompanied by changes in the dynamic balance of brain networks, and microstates provide a part of the functional brain network basis for us to identify these changes. These changes in temporal dynamic parameters in the early stages may suggest the clinical traits and functional recovery in these patients. In addition, we also measured the spontaneous activities of the upper limbs of the two upper limbs of the patients with CI by using actiwatches, which validates our perspective on cerebral infarction patient identification and assessment that choosing appropriate evaluation tools can provide multidimensional and interrelationship information.

## Data Availability

The raw data supporting the conclusions of this article will be made available by the corresponding author, without undue reservation.

## Footnotes

^1^ http://germangh.com
